# Potential Risk Factors for In-Hospital Mortality in Patients with Moderate-to-Severe Blunt Multiple Trauma Who Survive Initial Resuscitation

**DOI:** 10.1155/2018/6461072

**Published:** 2018-11-22

**Authors:** Neslihan Yucel, Tuba Ozturk Demir, Serdar Derya, Hakan Oguzturk, Murat Bicakcioglu, Funda Yetkin

**Affiliations:** ^1^Department of Emergency Medicine, Inonu University, School of Medicine, Malatya, Turkey; ^2^Department of Anesthesiology and Reanimation, Inonu University, School of Medicine, Malatya, Turkey; ^3^Department of Infectious Disease and Clinical Microbiology, Inonu University, School of Medicine, Malatya, Turkey

## Abstract

**Introduction:**

The aim was to identify risk factors that influence in-hospital mortality for patients with moderate-to-severe blunt multiple trauma (BMT) who survive initial resuscitation.

**Methods:**

The prospective study involved 195 adult patients with BMT who were admitted to a referral hospital's emergency department (ED) between May 1, 2015, and May 31, 2016.

**Results:**

Forty-three (22%) of the 195 patients died in hospital. Multivariate analysis identified low blood pH (odds ratio [OR] 6.580, 95% confidence interval [CI] 1.12-38.51), high serum lactate level (OR 1.041, 95% CI 1.01-1.07), high ISS (OR 1.109, 95% CI 1.06-1.16), high APACHE II score (OR 1.189, 95% CI 1.07-1.33), traumatic brain injury (TBI) (OR 4.358, 95% CI 0.76-24.86), severe hemorrhage (OR 5.314, 95% CI 1.07-26.49), and coagulopathy (OR 5.916, 95% CI 1.17-29.90) as useful predictors of acute in-hospital mortality. High ISS (OR 1.047, 95% CI 1.02-1.08), TBI (OR 8.922, 95% CI 2.57-31.00), sepsis (OR 4.956, 95% CI 1.99-12.36), acute respiratory distress syndrome (ARDS) (OR 8.036, 95% CI 1.85-34.84), respiratory failure (OR 9.630, 95% CI 2.64-35.14), renal failure (OR 74.803, 95% CI 11.34-493.43), and multiple organ failure [MOF] (OR 10.415, 95% CI 4.48-24.24) were risk factors for late in-hospital mortality. High Glasgow Coma Scale (GCS) was a good predictor for survival at 2, 7, and 28 or more days of hospitalization (OR 0.708 and 95% CI 0.56-0.09; OR 0.835 and 95% CI 0.73-0.95; OR 0.798 and 95% CI 0.71-0.90, resp.).

**Conclusion:**

Several factors signal poor short-term outcome for patients who present to the ED with moderate-to-severe BMT: low blood pH, high serum lactate level, presence of TBI, severe hemorrhage, coagulopathy, organ failure (respiratory, renal, and MOF), and ARDS. For this patient group, ISS and APACHE II scores might be helpful for stratifying by mortality risk, and GCS might be a good predictor for survival.

## 1. Introduction

Despite significantly improved traffic safety and occupational safety and despite advances in prehospital and in-hospital management, multiple trauma remains the most frequent cause of death and disabilty worldwide in persons younger than 40 years [1, 2]. Trauma-related death is a subject of great interest in the global literature and can occur in the prehospital period, on admission to the emergency department (ED), or during hospitalization. The issue of identifying reasons for death after trauma has been addressed by many researchers and will continue to be investigated until there is a decrease in deaths from potentially preventable and treatable causes [3–5]. Various factors, such as age, severity of injury, site of injury, time to definitive care, quality of care, and presence of coagulopathy and hemorrhagic shock, are known to affect mortality after multiple trauma [2, 4, 6]. Mortality and morbididty can be reduced by educating society about first aid for multiple trauma and by better educating medical staff regarding treatment [7, 8]. Apart from cases of immediate death after trauma, many predictors of mortality in this patient group are controllable or treatable at the scene, upon admission to the ED, and during hospitalization.

The aim of this study was to identify risk factors that influence in-hospital mortality for patients with moderate-to-severe blunt multiple trauma (BMT) and that can be used to develop effective prevention and comprehensive management strategies.

## 2. Materials and Methods

**Figure 1:**
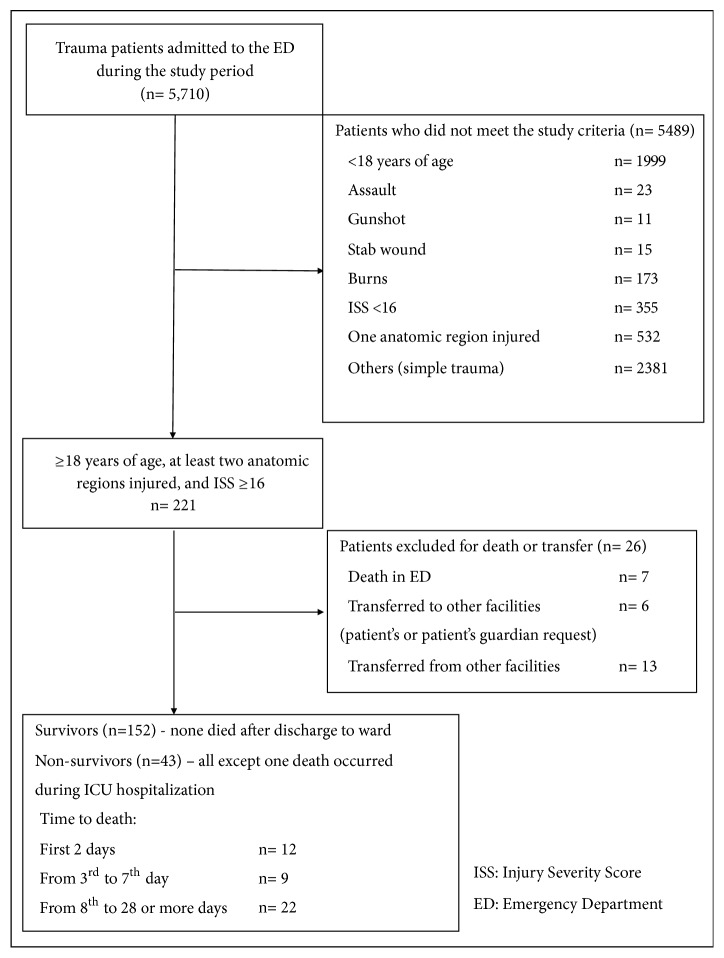
Flow chart depicting the total trauma patients admitted during the study period and the patients ultimately enrolled after applying the various inclusion and inclusion criteria.

The study was conducted in the ED of Inonu University Hospital, Turkey. The university's Institutional Review Board approved the study design, and patients or patients' relatives provided written consent. The participants were 195 adult patients with multiple trauma who had fallen from heights or had motor vehicle or pedestrian accidents and had been directly admitted to our ED from their accident scene between May 1, 2015, and May 31, 2016. The inclusion criteria were ≥18 years of age, BMT, at least two anatomic regions injured, and injury severity score (ISS) ≥16. Patients were also excluded if they were transferred to our ED from another hospital or transferred to another institution or if they died during ED management. All patients were followed up until hospital discharge or in-hospital death.  Figure 1 depicts the number of total traumas during the study period, patients excluded and included, and deaths among those ultimately enrolled.

Standard study forms were prepared for recording patient data. In each case, data were collected daily, beginning with admission to the ED. Once a patient was hospitalized, data were collected by his/her doctor in the ICU and surgical ward.

Upon arrival at the ED, each patient was monitored and the pulse rate, arterial blood pressure, fever, respiratory rate, and oxygen saturation were recorded on the study form. The patient was examined quickly and Glasgow Coma Scale (GCS) score was determined [9]. Two large vessels were catheterized and blood samples were drawn to determine blood type and crossmatch and to measure hemoglobin, hematocrit, platelet count, activated partial thromboplastin time (aPTT), international normalized ratio (INR), glucose, lactate, renal function indicators, liver enzymes, arterial blood gases, and electrolytes. After these procedures, resuscitation was conducted following Advanced Trauma Life Support (ATLS) principles and the patient underwent diagnostic and therapeutic procedures according to existing protocol [10]. As well, FAST examination was routinely performed for each trauma patient.

The documentation for each patient included detailed information on demographics (i.e., age, sex, and comorbidities), mechanism of injury, duration of hospital and ICU stay, surgery, pre- and in-hospital management, vital signs and laboratory findings on admission, interventions during admission and hospitalization, course in the ICU and surgical ward, and outcome. Any occurrences of organ failure and complications were recorded daily during follow-up after the patient was hospitalized. In each case, ISS was determined during ED admission [11] and Acute Physiology and Chronic Health Evaluation II (APACHE II) score was determined during ICU admission [12].

Multiple trauma was defined as presence of two or more system injuries. During ED evaluation, diagnosis and injury pattern were established according to laboratory, radiological, and physical examination findings. Most patients had head trauma. Any individual with signs and symptoms of this based on physical findings was evaluated as a head injury. Presence of severe or moderate TBI was also assessed at ED admission. This was diagnosed based on GCS ≤12 and some patients also had internal brain injury and skull fracture on computed tomography or had concussion at admission [13].

Severe hemorrhage (stage 3 or 4 hemorrhagic shock) was evaluated in the ED as well. This was identified based on vital signs, capillary refill time, urine output, and consciousness and required transfusion of packed red blood cells (RBCs) [10]. If a patient was diagnosed with hemorrhagic shock and required a massive transfusion protocol from the time of ED presentation, damage control resuscitation was initiated. This involved replacement of >4 units of RBCs within 1 h or 10 units of RBCs within 24 h, with the aim of achieving approximately 1:1:1 ratio of packed RBCs, fresh frozen plasma/cryoprecipitate, and platelets.

In accordance with previous studies, during ED admission, acute traumatic coagulopathy was diagnosed as (1) aPTT/prothrombin time (PT) greater than 1.5 x normal values, (2) aPTT greater than 60 s, or (3) INR greater than 1.5 and platelet count <100x 109/L [14, 15]. Organ failure during ICU admission or hospitalization was defined according to previous studies [16–19]. To be identified as organ failure (and to exclude patients with transient organ dysfunction), the criteria had to be present for 3 consecutive days. Neurologic failure was excluded from the study because most of the patients had head trauma. Sepsis, acute respiratory distress syndrome (ARDS), and infection were defined according to previous studies [19–21]. Renal replacement therapy was defined as any form of hemodialysis or hemofiltration alone or in combination.

### 2.1. Statistical Analysis

Findings were analyzed with patients categorized in survivor and nonsurvivor groups. The software package PASW (Predictive Analytics SoftWare) for Windows version 18.0 (SPSS, Inc., Chicago, IL, USA) was used for statistical analysis. Descriptive statistics were reported, including mean, standard deviation, and frequency. Categorical data were analyzed using the chi-square or Fisher's exact test. Continuous data were analyzed using the unpaired* t*-test or Mann-Whitney* U* test, depending on whether the data were normally distributed and occurrence of death by 2, 7, and 28 or more days of hospitalization. Associations between risk factors and death were first tested in a series of univariate models. The independent variable with the most significant* p *value (at a threshold of* p*≤0.1) was chosen as the first variable to be added to the multivariate model. For subsequent iterations, all remaining variables were individually added to the model, and the variable with the most significant result was retained. Additional variables were added as long as the* p* value of the backward-stepwise test was less than 0.05. Variables that were not applied in this model were used in a forward-stepwise test. Estimated odds ratios (ORs) and their corresponding 95% confidence intervals (CIs) were reported.

## 3. Results

Table 1 lists patient characteristics, other case details, and results of univariate analysis. The patients were 143 males and 52 females with median age of 49 years (range: 18 to 85 years). Of the total 195 patients, 149 (76%) were transferred to ICU and 149 (76%) underwent surgery. Emergent surgery was required in 53 cases (27% of all patients). The nonsurvivors had significantly higher mean APACHE II score and mean ISS and lower mean GCS than the survivors (p<0.0001 for all). During the study period, 43 (22%) of the patients died in hospital.

Table 2 shows the causes of death from admission to the 7th day of hospitalization (acute- and early-phase deaths) and diagnosis on admission from the 8th to the 28th day or later (late-phase deaths). Most of the deaths occurred within one month (36 patients, 84% of all deaths).

Table 3 summarizes patient results for vital signs and laboratory findings while in the ED. The nonsurvivors had significantly lower mean arterial pressure and significantly higher mean heart rate than survivors (*p*<0.0001 for both). Mean values for blood pH, bicarbonate level, arterial oxygen pressure, and hemoglobin level were significantly lower in the nonsurvivor group (*p*<0.0001,* p*<0.0001,* p*=0.006, and* p*<0.001, resp.). The nonsurvivor group's means for serum lactate and glucose were significantly higher than those of the survivor group (*p*<0.0001 for both). When the 195 patients were stratified by severe hemorrhage and coagulopathy, mean lactate level was significantly higher in the subgroup with severe hemorrhage and the subgroup with coagulopathy (*p*<0.0001 for both).

The results for injury pattern and diagnosis during ED evaluation are shown in Table 4. Most common were extremity injury (*n=*168, 86% of all patients), thoracic injury (*n=*103, 53%), head injury (*n=*100, 51%), and maxillofacial injury (*n=*39, 20%). Frequencies of severe TBI, pneumothorax, hemorrhagic shock, and coagulopathy were all significantly higher in the nonsurvivor group than in the survivor group (p<0.0001, p=0.008, p<0.0001, and p<0.0001, resp.). Hemorrhagic shock was identified in 31 (38%) of the 81 patients with TBI, and more of those individuals died than survived (20 versus 11;* p=*0.004).

Fifteen (8%) of the 195 patients were diagnosed with coagulopathy during ED admission, and a higher proportion of the nonsurvivors had coagulopathy compared to the survivors (p<0.0001). Eleven (73%) of the 15 patients who had coagulopathy during ED admission developed multiple organ failure (MOF), and all 15 (100%) developed complications. Frequencies of MOF and complications were significantly higher among the patients with coagulopathy (p<0.001 and p<0.0001, resp.).

Table 5 summarizes the results for complications, organ failure, and specific interventions during ED admission and hospitalization. Ninety (46%) of the 195 patients had complications, and one or more complications occurred in 56 (29%) of the survivors and 34 (17%) of the nonsurvivors. The most frequent complications among survivors were pneumonia, sepsis, surgery site infection, and urinary tract infection. Nonsurvivors had higher frequencies of pneumonia, sepsis, and ARDS than survivors (*p*<0.0001,* p*<0.0001, and,* p*=0.002, resp.). Eighty-six (58%) of the 149 patients were admitted to ICU developed complications, whereas only four (9%) of the 46 admitted to the surgical ward developed complications (*p*<0.0001).

Organ failure occurred in 67 (34%) of the 195 total patients, and 58 of those individuals (30% of the 195 total) developed MOF. There were higher frequencies of organ failure and MOF among the nonsurvivors compared to the survivors (*p*<0.0001 for both). All except one of the patients who had MOF were admitted to ICU. As expected, the subgroup of patients who developed MOF had significantly higher frequencies of complications than those who did not (p<0.0001).

Nonsurvivors had higher frequencies of mechanical ventilation, tube thoracostomy, renal replacement therapy, vasoactive drug infusion, packed RBCs transfusion, fresh frozen plasma transfusion, and platelet transfusion (*p*<0.0001,* p*=0.004,* p*<0.0001,* p*<0.0001,* p*<0.0001,* p*<0.0001,* p*<0.0001, and* p*<0.0001, resp.). Eighty-nine (60%) of the 149 patients admitted to ICU required mechanical ventilation, whereas only three (7%) of the 46 admitted to the surgical ward required this support (*p*<0.0001). Thirteen (9%) of the 152 survivors needed rehabilitation for permanent motor and neurologic consequences of BMT.

Table 6 lists the descriptive statistics for GCS, ISS, APACHE II, serum lactate, TBI, coagulopathy, hemorrhage, and ICU admission among the patients who developed complications (*n=*90) and MOF (*n=*58) and their respective counterpart groups. Both these subgroups had significantly higher mean APACHE II score and ISS and lower mean GCS than their respective counterpart groups without complications and without MOF (*p*<0.0001 for all). These subgroups also had significantly higher mean serum lactate levels than their counterparts (*p*<0.0001 for both). Frequencies of complications and MOF were significantly higher among the subgroups with TBI and hemorrhage, respectively, than among the subgroups without TBI and hemorrhage (*p*<0.0001 for all). In addition, frequencies of complications and MOF were significantly higher in the subgroup with coagulopathy than in the subgroup without coagulopathy (*p*<0.0001 and* p*<0.001, resp.). Frequencies of complications and MOF were also significantly higher, respectively, among patients who required ICU admission than among those who did not require ICU admission (*p*<0.0001 for both).

The logistic regression results for the mortality risk factors identified and their influences on death at 2, 7, and 28 or more days of hospitalization are listed in Table 7. Multivariate analysis identified low blood pH (OR 6.580, 95%CI 1.12-38.51), high serum lactate (OR 1.041, 95%CI 1.01-1.07), high ISS (OR 1.109, 95%CI 1.06-1.16), high APACHE II score (OR 1.189, 95% 1.07-1.33), TBI (OR 4.358, 95%CI 0.76-24.86), severe hemorrhage (OR 5.314, 95%CI 1.07-26.49), and coagulopathy (OR 5.916, 95%CI 1.17-29.90) as useful predictors of acute in-hospital mortality. Low blood pH (OR 5.664, 95%CI 1.65-19.40), high serum lactate (OR 1.025, 95%CI 1.00-1.05), high ISS (OR 1.089, 95%CI 1.05-1.13), high APACHE II score (OR 1.172, 95%CI 1.07-1.26), TBI (OR 3.789, 95%CI 1.17-12.24), severe hemorrhage (OR 5.370, 95%CI 1.76-16.7), coagulopathy (OR 7.455, 95%CI 1.95-26.53), respiratory failure (OR 20.380, 95%CI 3.26-127.53), renal failure (OR 6.745, 95%CI 2.36-19.31), and MOF (OR 3.366, 95%CI 1.24-9.17) were risk factors for early in-hospital mortality. High ISS (OR 1.047, 95%CI 1.02-1.08), TBI (OR 8.922, 95%CI 2.57-31.00), sepsis (OR 4.956, 95%CI 1.99-12.36), ARDS (OR 8.036, 95%CI 1.85-34.84), respiratory failure (OR 9.630, 95%CI 2.64-35.14), renal failure (OR 74.803, 95%CI 11.34-493.43), and MOF (OR 10.415, 95%CI 4.48-24.24) were risk factors for in-hospital mortality at 28 or more days. High GCS was a good predictor of survival at 2, 7, and 28 or more days of hospitalization (OR 0.708 and 95%CI 0.56-0.09; OR 0.835 and 95%CI 0.73-0.95; OR 0.798 and 95%CI 0.71-0.90, resp.).

## 4. Discussion

Blunt trauma is increasing in frequency, especially in developing countries. Younger patients are more likely to sustain these injuries, usually due to automobile accidents or falls from height. Our findings for injury mechanism and age were similar to those of previous studies [1, 2, 22]. MacLeod et al. [4] found that increasing age was an independent and untreatable indicator for mortality. However, there was no statistically significant relationship between mortality and age or injury mechanism in our study.

Regarding the BMT patients in our study at a level 1 trauma center, the in-hospital death rate was 22% and most deaths occurred within the first month after injury. This is in accordance with previous studies. Paffrath et al. [23] investigated 45,350 moderate-to-severe BMT patients in Germany and reported an overall hospital mortality rate of 20.4%. MacKenzie et al. [24] reported 21.3% in this group. Prin et al. [25] studied patients with BMT who were admitted to the ICU after in-hospital complications and recorded 16.9% hospital mortality. However, the literature provides no standards of reference for expected rates of morbidity and mortality among patients with BMT. Authors from different world regions have reported different mortality rates for trauma patients. There are various potential reasons for this, including age, severity or site of injury, prehospital and hospital care, mechanism of injury, misdiagnosis, and lack of facilities for interventions.

Given that it is difficult to predict death in patients with multiple trauma, several severity scoring systems have been developed to gauge severity of trauma and predict death risk in this group: GCS, ISS, AIS, APACHE II, revised trauma score, trauma and injury severity score, and the new injury sverity score [9, 11, 12, 26–28]. Many researchers have investigated which scoring system is superior for determining severity of injury and mortality in prehospital and hospital settings [29, 30], and results have differed. Most authors have identified higher ISS as a risk factor for mortality in BMT patients [23, 24]. Our univariate analysis showed that nonsurviving patients with BMT had higher ISS and APACHE II and lower GCS scores than survivors. Using the multivariate model, we identified high ISS as an independent risk factor for in-hospital mortality at 2, 7, and 28 or more days' hospitalization in this patient group. Also, similar to previous studies, our univariate analysis revealed that high APACHE II score was associated with MOF and complications, and the multivariate model identified this variable as an independent predictor of in-hospital mortality at 2 and 7 days after injury [12]. As would be expected, we observed a negative correlation between high GCS and in-hospital mortality at 2, 7, and 28 or more days of hospitalization. Our results clearly identified high GCS at admission as a good predictor of survival. Univariate analysis also revealed more frequent organ failure and complications among patients with high ISS and low GCS score.

Acidosis occurs secondary to rapid blood loss in patients with multiple trauma and develops as a result of tissue damage or hypoperfusion. More recently, biomarkers pH, lactate, and base excess have been used to determine tissue hypoperfusion, to determine the need for intervention to help improve outcomes, and to predict mortality [31–33]. Several studies have reported that inability to respond to resuscitation and normalize acidosis is associated with death [34, 35]. Blood lactate levels are recommended for evaluating the severity of illness, predicting mortality, and response to resuscitation in critical patients [36]. Several studies have shown that, for patients with multiple trauma, elevated serum lactate on admission is related to higher mortality and need for transfusion of blood products [31–33, 35–37]. In contrast, Freites et al. [38] observed no correlations between admission lactate level or lactate clearance and mortality in patients treated for multiple trauma. Manikas et al. [34] did detect significant relationships between blood lactate level and development of MOF and complications among patients with multiple trauma. Our results were smilar to previous findings. In addition, the multivariate model identified low blood pH and high serum lactate level during ED admission as useful predictors of in-hospital mortality at 2 and 7 days. Lactate level may be useful to monitor as a gauge of response to resuscitation and for predicting severity of illness in the ED.

The main causes of death in BMT patients who arrive at hospital alive are primary severe head injury, followed by hypovolemic shock, sepsis, and MOF [1–3]. In our study, 27 (14%) of the 51 patients with severe TBI eventually died, and univariate analysis revealed that severe TBI was associated with mortality. This is in accordance with other studies. Indeed, when not associated with hemorrhage, mortality has been found to depend on the severity of the initial intracranial injury [39]. The combination of TBI and shock has also been found to be responsible for very high mortality as well as worsening neurological prognosis in patients with multiple trauma [40]. Lichtvelt and colleagues identified presence of isolated neurological damage as a risk factor for death, and they found that severe head injury and hemorrhage were the most important risk factors for death in the first 24 hours after accident [41]. Our results were similar. The multivariate model identified TBI as an independent risk factor for in-hospital mortality at 2, 7, and 28 or more days in patients with moderate-to-severe BMT. Additionally, univariate analysis showed that TBI was a risk factor for developing complications and MOF.

Fifty-two (27%) of our BMT patients had severe hemorrhage at the time of ED admission, and half of those patients died. Univariate analysis revealed an association between hemorrhage and mortality, as reported by MacLeod et al. [42]. Our multivariate model identified severe hemorrhage as a predictor of in-hospital mortality at 2 and 7 days. There have been substantial improvements in hemorrhage management, such as implementation of the ATLS concept in the prehospital setting and ED, hospital diagnostic techniques, and treatment strategies (interventional radiologic procedures, damage control resuscitation, and damage control surgery); however, uncontrolled hemorrhage remains the leading cause of early preventable death in cases of severe trauma [1, 2, 43]. Among our BMT patients, we observed that when hemorrhage was accompanied by TBI, coagulopathy, and hyperlactatemia at time of admission, death occurred despite all interventions. Additionally, our univariate analysis revealed that hemorrhage was associated with development of complications and MOF. Presence of hemorrhagic shock is a predictor of poor outcome in trauma patients, and the volume of hemorrhage is associated with outcome [44]. As the amount of blood loss increases, so do resuscitation requirements and physiologic derangements, including hypotension and acidosis. Hypotension noted in the field or during ED evaluation is associated with mortality [25, 42, 45]. Also, hypotensive patients require early volume replacement therapy, including blood and blood products, and the amount of fluid resuscitation required is related to development of coagulopathy, complications including MOF, and infectious processes such as sepsis and pneumonia [42, 44]. In the present study, we observed a significantly higher frequency of hypotension among the nonsurviving BMT patients, and univariate analysis revealed that nonsurvivors also had higher frequencies of need for transfusion of blood and blood products.

Coagulopathy after trauma has mainly been attributed to the amount of outright bleeding that occurs and subsequently to trauma-hemorrhage-associated dilution phenomena from intravenous fluid therapy, massive blood transfusion, progressive hypothermia, and acidosis [46, 47]. In accordance with the findings of Brohi and colleagues [47], we detected coagulopathy in 8% of BMT patients who had received only minimal resuscitation in the field. As well, coagulopathy was associated with mortality, which was consistent with other studies in literature [14, 42, 43]. The multivariate model identified coagulopathy as a risk factor for in-hospital mortality at 2 and 7 days. Additionally, univariate analysis showed that BMT patients who presented with coagulopathy were at high risk for developing organ failure and complications. Several studies have demonstrated that elevated aPTT/INR combined with low hemoglobin level is one of the most important predictors of outcome [14, 42, 47]. Our univariate analysis findings were in accordance with this.

Multiple organ failure remains a major cause of morbidity and late postinjury death [2, 48]. Indeed, the incidences of MOF and related mortality vary depending on the type of the trauma, the number of organs involved, and whether the patient develops sepsis. Occurrence of MOF after blunt trauma is more common than after penetrating trauma [48, 49]. Trauma patients who survive the early phase typically receive optimal initial management and are admitted to ICUs where they receive expensive and high-quality care. This situation results in longer ICU stays, which can involve MOF and complications such as sepsis. Fröhlich and colleagues [49] reported a 33% incidence of MOF in patients with ISS of 16 or higher. Our MOF incidence was similar. Respiratory dysfunction is the main contributor to early MOF, followed by cardiovascular decompensation and renal and hepatic failure [17, 50, 51]. In accordance with previous studies, we found that the respiratory system was most commonly affected, followed by the cardiovascular system. The multivariate model identified MOF, respiratory failure, and renal failure as risk factors for in-hospital mortality at 7 and 28 or more days. Univariate analysis also identified low pO_2_ and low oxygen saturation on ED admission (which may signal early respiratory failure and need for early mechanical ventilation) as being associated with mortality. Mechanical ventilation is frequently required when managing respiratory failure. With the exception of BMT patients who are intubated for airway protection, alternatives to mechanical ventilation may be explored in some cases to reduce the risk of associated complications.

Advances in trauma management have increased the survival rate of patients with multiple trauma but have rendered them vulnerable to nosocomial infections and other complications during the course of in-hospital treatment [52]. Complications may arise from the direct effects of the injury or from side effects of therapy. Further, the improved ability to keep severely injured trauma patients alive has resulted in increased incidence of complications. As noted in previous studies, for our BMT patients, we found that development of hospital course complications was more common among those admitted to ICU than among those hospitalized without ICU admission, and we observed 17% overall hospital mortality [25]. Prin et al. [25] reported that the most common complications among patients admitted to the ICU were pneumonia, urinary tract infection, and ARDS. In our study of 195 BMT patients, the most common complications were organ failure followed by pneumonia, sepsis, and surgery site infection. Types and development of complications may be associated with severity of injury, site of injury, misdiagnosis, and interventions on admission and after hospitalization, such as mechanical ventilation, transfusion of blood products, surgery, and other invasive procedures. Ingraham et al. found that pneumonia, ARDS, sepsis, and surgery site infection were risk factors for hospital mortality [53]. Our univariate analysis showed that pneumonia, sepsis, and infection after orthopedic surgery were related to mortality. Multivariate analysis identified sepsis as a predictor of in-hospital mortality at 28 or more days. Although the incidence of posttraumatic sepsis in the hospital has decreased over the past two decades, the mortality risk remains high for trauma patients who develop sepsis [54].

Trauma is one of the most important risk factors for the development of ARDS; this syndrome occurs in 12% to 25% of trauma patients and is associated with higher rates of hospital mortality and morbidity [55]. Known predictors of ARDS in patients with multiple trauma are high ISS and APACHE II score, age ≥65 years, presence of hemorrhagic shock, pulmonary contusion, presence of a femur fracture, and massive RBC transfusion [56]. In our study, ARDS occurred in 13 (7%) of the patients with moderate-to-severe BMT, and the nonsurvivors had a significantly higher frequency of this syndrome. Additionally, the multivariate model identified ARDS as a predictor of in-hospital mortality at 28 or more days.

This study had some limitations in that it was performed at a single center, involved a small sample, and focused on blunt trauma exclusively. Also, the data yield information that pertains most strongly to the local population (i.e., Malatya, Turkey).

## 5. Conclusion

Since most patients with moderate-to-severe BMT are first evaluated in the ED, it is important that emergency physicians can accurately assess mortality risk for such individuals at presentation. Our results point to a list of potentially important risk factors for acute, early, and late mortality in patients with moderate-to-severe BMT who are admitted to the ED, ICU, and/or the surgical ward: low blood pH, high serum lactate, coagulopathy, TBI, severe hemorrhage, high APACHE II score, high ISS, low GCS, organ failure, and complications. While hemorrhage, high serum lactate, low pH, and coagulopathy were identified as potentially treatable predictors of acute- and early-phase mortality, complications and organ failure (respiratory, renal, and multiorgan) are preventable and treatable predictors of early and late mortality in patients with moderate-to-severe BMT. We also found that ISS and TBI are independent untreatable predictors of acute, early, and late mortality and that high GCS at admission is a good predictor of survival in this group. Additionally, our findings show that sepsis and ARDS are strong predictors of late mortality for these patients.

## Figures and Tables

**Table 1:** Patients demographics, findings on initial evaluation, and hospitalization.

	**Survivors** **(*n*=152, 88**%**)**	**Nonsurvivors** **(*n*=43, 22**%**)**	**All patients** **(*n*=195)**	***p*** **∗**
**Age (years)**	44±18	51±22	45±19	**0.068**
**Sex (n[**%**])**				0.212
Female	38 (25%)	14 (33%)	52 (27%)	
Male	114 (75%)	29 (67%)	143 (73%)	
**Comorbidity (n [**%**])**	12 (8%)	5 (12%)	7 (4%)	0.312
**Mechanism of injury (n[**%**])**				0.101
Motor vehicle accident	70 (46%)	11 (26%)	81 (42%)	
Pedestrian accident	37 (23%)	15 (35%)	52 (27%)	
Fall from height	39 (25%)	14 (33%)	53 (27%)	
Other (e.g., motorcycle, bicycle, tractor)	6 (4%)	3 (7%)	9 (5%)	
**GCS **	13±3	8±4	12±4	**0.0001**
**ISS **	30±13	51±17	35±16	**0.0001**
**APACHE II **	10±8	34±9	15±13	**0.0001**
**Surgery (n[**%**])**	116 (76%)	33 (77%)	149 (76%)	0.565
Emergent surgery	30 (20%)	23 (53%)	53 (27%)	**0.0001**
Urgent surgery	97 (64%)	18 (42%)	115 (59%)	**0.008**
**Hospitalization of the patients (n[**%**])**				**0.0001**
Surgical wards	45 (30%)	1 (2%)	46 (24%)	
ICU	107 (70%)	42 (98%)	149 (76%)	
**Duration of hospital stay (days)**	15±17	15±17	15±17	0.175
**Duration of ICU stay (days) **	10±16	15±17	11±17	**0.003**

GCS: Glasgow Coma Scale; ISS: Injury Severity Score; APACHE II: Acute Physiology and Chronic Health Evaluation II; ICU: Intensive care unit.

*∗p *values for comparisons between the surviving and nonsurviving groups. *∗∗*For example, bladder, kidney, ureter, maxillofacial, tissue defect, and vascular injury

**Table 2:** Causes of death in the acute and early phases after trauma and diagnosis on admission between 8 and 28 days or later for the 43 deaths.

	**Deaths admission and 2 days' hospitalization (acute phase)** **(n=12, 28**%**)**	**Deaths between 3 and 7 days (early phase)** **(n=9, 21**%**)**	**Deaths between 8 to 28 days or more (late phase)** **(n=22, 51**%**)**	**Total deaths** **(n=43)**
**Traumatic brain injury**	3 (7%)	3 (7%)	10 (23%)	16 (37%
**Severe hemorrhage**	2 (5%)	2 (5%)	2 (5%)	6 (14%)
**Traumatic brain injury and severe hemorrhage**	7 (16%)	3 (7%)	10 (23%)	20 (47%)
**Pulmonary embolism**	-	1 (2%)	-	1 (2%)

**Table 3:** Patients' vital signs and laboratory findings during emergency admission.

	**Survivors** **(n=152, 88**%**)**	**Nonsurvivors** **(n=43, 22**%**)**	**All patients** **(n=195)**	***p*** **∗**
**Vital signs**				
Mean arterial pressure (mmHg)	90±17	69±25	85±21	**0.0001**
Heart rate (beats/min)	82±14	100±28	86±20	**0.0001**
Respiratory rate (breaths/min)	19±4	17±11	19±6	0.223
Body temperature (°C)	36.5±0.3	36.4±0.3	36.5±0.3	0.085
**Laboratory findings**				
pH	7.387±0.1	7.273±0.2	7.362±0.1	**0.0001**
pO_2_ (mmHg)	82±25	71±29	80±27	**0.006**
pCO_2_ (mmHg)	35±7	38±14	36±9	0.080
HCO_3_ (mEq)	22±4	18±5	21±4	**0.0001**
O_2_ saturation (%)	92±11	82±23	90±15	**0.015**
Lactate (mg/dL)	27±17	43±22	30±19	**0.0001**
White blood cell count (10^3^ cells/mm3)	16.2±7.1	16.8±6.1	16.3±6.1	0.432
Hemoglobin (g/dL)	13.1±2.3	11.5±2.6	12.7±2.5	**0.001**
Hematocrit (%)	40±6	35±7	38±7	**0.001**
Platelet count (10^3^ cells/mm3)	246±83	241±101	245±87	0.635
aPTT (sec)	32±32	42±49	34±36	**0.024**
International normalized ratio	1.1±0.2	1.3±0.6	1.3±0.6	**0.029**
Serum glucose (mg/dL)	154±60	201±88	146±70	**0.0001**
Blood urea nitrogen (mg/dL)	16±5	16±4	16±5	0.383
Serum creatinine (mg/dL)	0.8±0.5	0.9±0.3	0.9±0.5	**0.019**
Serum alanine aminotransferase (IU)	80±129	111±250	87±163	0.737
Serum aspartate aminotransferase (IU)	91±115	126±203	99±140	0.223
Serum lactate dehydrogenase (IU)	518±277	591±385	591±385	0.209

aPTT: activated partial thromboplastin time.

*∗p *values for comparisons between the surviving and nonsurviving groups.

**Table 4:** Patterns of injury and diagnosis during ED evaluation according to physical examination, laboratory, and radiological findings for the 195 patients.

	**Survivors** ** (*n=*152, 88**%**)**	**Nonsurvivors** **(*n=*43, 22**%**)**	**All patients** **(*n=*195)**	***p*** **∗**
**Head injury (n **[%]**)**	**62 (41**%**)**	**38 (88**%**)**	**100 (**%**51)**	**0.0001**
Severe TBI	**24 (16**%**)**	**27 (63**%**)**	**51 (26**%**)**	**0.0001**
Moderate TBI	**21 (14**%**)**	**9 (21**%**)**	**30 (15**%**)**	0.182
Subarachnoid bleeding	26 (17%)	23 (53%)	49 (25%)	**0.0001**
Subdural/epidural hematoma	20 (13%)	24 (56%)	44 (23%)	**0.0001**
Intraparenchymal bleeding	15 (10%)	5 (12%)	20 (10%)	0.462
Contusion	10 (6%)	8 (19%)	18 (9%)	**0.023**
Diffuse axonal injury	8 (4%)	6 (14%)	14 (7%)	0.060
Fracture	29 (19%)	18 (42%)	47 (24%)	**0.002**
**Extremity injury (n **[%]**)**	**132 (87**%**)**	**36 (84**%**)**	**168 (86**%**)**	0.381
Pelvis fracture	42 (28%)	12 (28%)	52 (27%)	0.556
Femur fracture	40 (26%)	9 (21%)	49 (25%)	0.307
Others bone fractures	98 (64%)	31 (72%)	129 (66%)	0.228
Amputation	5 (3%)	1 (2%)	6 (3%)	0.604
**Thoracic injury (n **[%]**)**	**76 (50**%**)**	**27 (63**%**)**	**103 (53**%**)**	0.095
Pulmonary contusion	47 (31%)	16 (37%)	63 (32%)	0.274
Haemothorax	29 (18%)	9 (21%)	38 (19%)	0.425
Pneumothorax	27 (18%)	16 (37%)	43 (22%)	**0.008**
** **Rib fractures	42 (28%)	17 (40%)	59 (30%)	0.096
**Maxillofacial injury (n **[%]**)**	**26 (17**%**)**	**13 (30**%**)**	**39 (20**%**)**	0.059
**Spinal injury (n **[%]**)**	**30 (20**%**)**	**9 (21**%**)**	**39 (20**%**)**	0.537
**Abdominal injury (n **[%]**)**	**20 (13**%**)**	**9 (21**%**)**	**29 (15**%**)**	0.153
Liver trauma	14 (9%)	8 (19%)	22 (11%)	0.079
Splenic injury	7 (5%)	4 (9%)	11 (6%)	0.204
Others	3 (2%)	0 (0%)	3 (2%)	0.472
**Genitourinary injury (n **[%]**)**	**2 (1**%**)**	**4 (9**%**)**	**6 (3**%**)**	**0.022**
**Vascular injury (n **[%]**)**	**6 (4**%**)**	**5 (12**%**)**	**11 (5**%**)**	0.067
**Severe hemorrhage** ^**α**^ ** (n **[%]**)**	**26 (17**%**)**	**26 (60**%**)**	**52 (27**%**)**	**0.0001**
**Coagulopathy on admission (n **[%]**)**	**6 (4**%**)**	**9 (21**%**)**	**15 (8**%**)**	**0.0001**

TBI: traumatic brain injury.

^*α*^Stage 3 and 4 hemorrhagic shockduring ED admission; *∗* represents *p* values for comparisons between the surviving and nonsurviving groups.

**Table 5:** Complications, organ failure, and specific interventions during ED admission and hospitalization.

	**Survivors** **(*n*=152, 88**%**)**	**Nonsurvivors** **(*n*=43, 22**%**)**	**All patients** **(*n*=195)**	***p*** **∗**
**Complications (n **[%]**)**	56 (37%)	34 (79%)	90 (46%)	**0.0001**
Pneumonia	27 (18%)	23 (53%)	50 (26%)	**0.0001**
Sepsis	18 (12%)	17 (40%)	35 (18%)	**0.0001**
Surgery site infection	18 (12%)	12 (28%)	30 (15%)	**0.012**
After orthopedic surgery	12 (8%)	9 (21%)	21 (11%)	**0.020**
After abdominal and/or genitourinary surgery	6 (4%)	3 (7%)	9 (5%)	0.315
Urinary tract infection	14 (9%)	8 (17%)	22 (11%)	0.079
ARDS	5 (3%)	8 (19%)	13 (7%)	**0.002**
Pulmonary emboli	4 (3%)	3 (7%)	7 (4%)	0.182
Compartment syndrome	0 (0%)	3 (7%)	3 (2%)	**0.010**
Others^*∗∗*^	7 (5%)	3 (8%)	10 (5%)	0.385
**Organ failure (n **[%]**)**	36 (24%)	31 (72%)	67 (34%)	**0.0001**
Respiratory	33 (22%)	31 (72%)	64 (33%)	**0.0001**
Cardiovascular	21 (13%)	31 (72%)	52 (27%)	**0.0001**
Hematologic	7 (5%)	26 (60%)	33 (17%)	**0.0001**
Liver	12 (8%)	19 (44%)	31 (16%)	**0.0001**
Renal	3 (2%)	24 (56%)	27 (14%)	**0.0001**
Multiorgan (two or more organs)	27 (18%)	31 (72%)	58 (30%)	**0.0001**
**Specific interventions (n **[%]**)**				
Mechanical ventilation	50 (28%)	42 (95%)	92 (43%)	**0.0001**
Noninvasive ventilation	36 (24%)	1 (2%)	37 (20%)	**0.0001**
Tube thoracostomy	17 (11%)	13 (30%)	30 (15%)	**0.004**
Packed red cell transfusion	69 (45%)	42 (98%)	111 (57%)	**0.0001**
Fresh frozen plasma transfusion	38 (25%)	41 (95%)	79 (41%)	**0.0001**
Platelet transfusion	18 (12%)	26 (60)	44 (23%)	**0.0001**
Antibiotherapy	143 (94%)	41 (95%)	184 (94%)	0.250
Vasoactive drug infusion	33 (22%)	37 (86%)	70 (36%)	**0.0001**
Tracheostomy	12 (8%)	14 (33%)	26 (13%)	**0.0001**
Renal replacement therapy	2 (1%)	11 (19%)	13 (4%)	**0.0001**
Pelvic embolization	2 (1%)	3 (7%)	5 (3%)	**0.072**

ARDS: acute respiratory distress syndrome; *∗p *values for comparisons between the surviving and nonsurviving groups; ^*∗∗*^for example, meningitis and intracranial abscess after brain surgery, seizure, arrhythmia, anoxic brain damage, diabetes insipidus, decubitus ulcer, and deep vein thrombosis.

**Table 6:** Descriptive statics for Glasgow Coma Score, Injury Severity Score, Acute Physiology and Chronic Health Evaluation II, serum lactate level, traumatic brain injury, coagulopthy, hemorrhage, and intensive care unit admission among patients who developed complications and multiorgan failure.

	**Complications **			**MOF**		
	**Present** **(*n=*90, 46**%**)**	**Absent** **(*n=*105)**	***p***	**Present** **(*n=*58, 29**%**)**	**Absent** **(*n=*137)**	***p***
Mean arterial pressure (mmHg)	79±23	91±17	0.0001	74±24	90±18	0.0001
GCS	10±4	14±3	0.0001	9±4	13±3	0.0001
ISS	42±17	29±13	0.0001	46±17	30±14	0.0001
APACHE II	24±13	8±8	0.0001	28±11	10±10	0.0001
Serum lactate (mg/dL)	38±22	24±14	0.0001	40±23	27±16	0.0001
Severe hemorrhage (n[%])	44 (85%)	8 (15%)	0.0001	32 (62%)	20 (38%)	0.0001
TBI (n[%])	57 (70%)	24 (30%)	0.0001	44 (54%)	37 (46%)	0.0001
Coagulopathy (n[%])	15 (100%)	0 (0%)	0.0001	11 (73%)	5 (33%)	0.001
ICU admission (n[%])	86 (58%)	63 (42%)	0.0001	57 (38%)	92 (62%)	0.0001
MOF	56 (97%)	2 (3%)	0.0001	-	-	-

Stage 3 and stage 4 hemorrhagic shock defined as severe hemorrhage. GCS: Glasgow Coma Score; ISS: Injury Severity Score; APACHE II: Acute Physiology and Chronic Health Evaluation II; TBI: traumatic brain injury; ICU: intensive care unit.

**Table 7:** Results of logistic regression analysis of risk factors for 2, 7, and 28 or more days in-hospital mortality for the patients with multiple trauma.

	**Between admission and 2 days in-hospital mortality** **(early phase)**		**Between 3 and 7 days in-hospital mortality** **(acute phase)**		**Between 8 and 28 or more days in-hospital mortality** **(late phase)**	
	OR (95% CI)	*p*	OR (95% CI)	*p*	OR (95% CI)	*p*
** pH ↓**	6.580 (1.12-38.51)	<0.001	5.664 (1.65-19.40)	0.006		
**Lactate ↑**	1.041 (1.01-1.07)	0.014	1.025 (1.00-1.05)	0.046		
**ISS**	1.109 (1.06-1.16)	<0.001	1.089 (1.05-1.13)	<0.001	1.047 (1.02-1.08)	0.003
**Higher GCS** **∗**	0.708 (0.56-0.09)	0.004	0.835 (0.73-0.95)	0.007	0.798 (0.71-0.90)	<0.001
**APACHE II**	1.189 (1.07-1.33)	0.002	1.172 (1.07-1.26)	<0.001		
**Traumatic brain injury**	4.358 (0.76-24.86)	0.098	3.789 (1.17-12.24)	0.026	8.922 (2.57-31.00)	0.001
**Severe hemorrhage** **∗** **∗**	5.314 (1.07-26.49)	0.042	5.370 (1.76-16.7)	0.003		
**Coagulopathy**	5.916 (1.17-29.90)	0.006	7.455 (1.95-26.53)	0.003		
**Respiratory failure**			20.380 (3.26-127.53)	0.001	9.630 (2.64-35.14)	0.001
**Renal failure**			6.745 (2.36-19.31)	<0.001	74.803 (11.34-493.43)	<0.001
**Multiple organ failure**			3.366 (1.24-9.17)	0.018	10.415 (4.48-24.24)	<0.001
**Sepsis**					4.956 (1.99-12.36)	0.001
**ARDS**					8.036 (1.85-34.84)	0.005

*∗*Positive predictor factor for survival. OR: odds ratios; 95% CI: 95% confidence intervals; *p*: *p* value. Stage 3 and stage 4 hemorrhagic shock defined as severe hemorrhage.

## Data Availability

The data used to support the findings of this study are available from the corresponding author upon request.
